# Repeated neonatal isoflurane exposures in the mouse induce apoptotic degenerative changes in the brain and relatively mild long-term behavioral deficits

**DOI:** 10.1038/s41598-019-39174-6

**Published:** 2019-02-26

**Authors:** Susan E. Maloney, Carla M. Yuede, Catherine E. Creeley, Sasha L. Williams, Jacob N. Huffman, George T. Taylor, Kevin N. Noguchi, David F. Wozniak

**Affiliations:** 1 Department of Psychiatry, Washington University School of Medicine, St. Louis, MO 63110 USA; 2 Department of Neurology, Washington University School of Medicine, St. Louis, MO 63110 USA; 3 Department of Psychology, State University of New York at Fredonia, Fredonia, NY 14063 USA; 4 Department of Psychology, University of Missouri – St. Louis, St. Louis, MO 63121 USA; 5 Taylor Family Institute for Innovative Psychiatric Research, Washington University School of Medicine, St. Louis, MO USA; 6 Intellectual and Developmental Disabilities Research Center, Washington University, St. Louis, MO USA

## Abstract

Epidemiological studies suggest exposures to anesthetic agents and/or sedative drugs (AASDs) in children under three years old, or pregnant women during the third trimester, may adversely affect brain development. Evidence suggests lengthy or repeated AASD exposures are associated with increased risk of neurobehavioral deficits. Animal models have been valuable in determining the type of acute damage in the developing brain induced by AASD exposures, as well as in elucidating long-term functional consequences. Few studies examining very early exposure to AASDs suggest this may be a critical period for inducing long-term functional consequences, but the impact of repeated exposures at these ages has not yet been assessed. To address this, we exposed mouse pups to a prototypical general anesthetic, isoflurane (ISO, 1.5% for 3 hr), at three early postnatal ages (P3, P5 and P7). We quantified the acute neuroapoptotic response to a single versus repeated exposure, and found age- and brain region-specific effects. We also found that repeated early exposures to ISO induced subtle, sex-specific disruptions to activity levels, motor coordination, anxiety-related behavior and social preference. Our findings provide evidence that repeated ISO exposures may induce behavioral disturbances that are subtle in nature following early repeated exposures to a single AASD.

## Introduction

There is continuing concern that exposing the developing brain to anesthetic agents and/or sedative drugs (AASDs) may induce neuropathologic effects leading to compromised cognitive functions. This concern is evidenced by a recent warning from the U.S. Food and Drug Administration, which stated that repeated or lengthy exposure to AASDs in children under the age of three, or in pregnant women during the third trimester, may affect children’s brain development^1,2^. However, the warning also importantly pointed out that based on animal and human studies, single or relatively brief exposures to these types of agents do not appear to pose substantial risk to the developing brain. Results from a meta-analysis conducted on epidemiological evidence linking anesthesia/surgery with poor neurodevelopmental outcomes in children showed a modestly elevated risk of adverse behavioral or developmental outcomes in children who were exposed to anesthesia/surgery early in childhood^3^. Subsequent research suggested longer, but not shorter, durations of anesthesia during a single exposure are associated with adverse outcomes^4,5^. Results from investigations into long-term academic and cognitive performance, as well as risk of mental disorders, developmental delay, and ADHD diagnoses following childhood surgery/anesthesia suggest that there may be subgroups vulnerable to adverse outcomes following early anesthesia exposure^6,7^. In addition, multiple, but not single, exposures to anesthesia have been linked to later learning disabilities and ADHD^8–11^, suggesting a particular risk to repeated exposures to anesthesia in early childhood. The fact that these drugs are often required for surgeries in young children or pregnant women underscores the necessity to identify conditions which pose risk for maldevelopment so that therapeutic strategies may be developed to mitigate adverse outcomes.

Animal studies have shown that one type of acute damage to the developing brain induced by AASDs is increased apoptotic neurodegeneration, which likely involves N-methyl-D-aspartate (NMDA) glutamate receptor antagonism or potentiation of γ-aminobutyric acid A (GABA_A_) receptors^12^. Recent animal studies have shown AASDs also impair neuronal function by disturbing timely axonal pruning and cues that promote accurate directional growth of axons during development^13,14^. Preclinical research has been valuable in defining the spatial and temporal patterns of the acute apoptotic damage, which is widespread and includes cell-type-specific patterns^1–3^. The degree to which specific brain areas are impacted depends on the developmental age each area is undergoing synaptogenesis, creating a window of vulnerability to AASD-induced apoptosis^15^. Neurons are most vulnerable to AASD-induced apoptosis from embryonic day (E)19 to postnatal day (P)14^16^ in rodents. While damage can be observed across many brain regions at any age in this window, stages of heightened vulnerability within distinct brain regions have been observed^17^.

Both rodent and non-human primate (NHP) models have been useful for elucidating long-term functional consequences of AASD-induced acute apoptotic damage and for identifying specific parameters that confer this risk (See Maloney *et al*.^16^ for full review). The majority of rodent studies devoted to characterizing the behavioral impairments related to AASD-induced apoptotic damage have involved treating mice or rats at P7 or later. This age corresponds to the peak of synaptogenesis within the rodent brain, and is analogous to the third trimester of pregnancy through the first few years of infancy in humans^18,19^. Only four rodent studies have involved behavioral analyses following exposure to an anesthetic agent at an age earlier than P7. Learning and memory deficits were reported in all four studies when mice or rats were tested at 1–2 months of age, and a longer-duration exposure to sevoflurane also produced disturbances in social interactions^20^. The behavioral effects of repeated exposures to an AASD at P7 and/or later have also been assessed in the rodent^21–27^. Only one out of eight of the repeated exposure studies failed to demonstrate functional impairments, which was likely due to a very short exposure duration (30 min)^28^. Studies involving the behavioral outcomes resulting from longer-duration developmental exposures to volatile anesthetics in rodents P7 or older, sometimes coupled with systemic sedative agents^29–35^, have also been conducted. Three out of ten of these studies reported no behavioral impairments, perhaps due to comparatively low concentrations of the anesthetic agent and the absence of any additional sedative agent^36–38^. Together, the findings from rodent studies on the functional consequences of developmental AASD exposure confirm that repeated and longer-duration exposures typically confer the greatest risk of behavioral impairments. However, there is scant evidence available regarding the impact of multiple exposures at earlier ages in the rodent, and very little work has been done to identify any sex-related effects in any murine models.

The relevance of rodent studies on the functional consequences of developmental AASD exposures has been confirmed by work using the infant NHP model. Of the five studies known to us, all have reported functional deficits later in life following infantile exposure to volatile general or systemic anesthetics. Behavioral perturbations include learning and memory deficits^39^, increased anxiety^40–42^, impaired motor reflexes, and altered social affiliation^42^. Results from NHP studies point out that the effects of early AASD exposure on levels of anxiety-like behaviors, motor performance, and social interactions have not been thoroughly examined in rodent models^16^. Thus, while the negative impact of repeated and longer-duration exposures on learning and memory performance have been fairly well documented during the middle stages of vulnerability (i.e., at P7) in rodents, the question of whether these same exposure parameters also produce deficits in other behavioral domains remains to be fully characterized. In addition, the few studies examining very early exposure to AASDs suggest this is a critical period for inducing long-term functional consequences, but the impact of repeated exposures at early ages has not yet been adequately assessed.

We have designed the present study to yield information with translational relevance to identifying aspects of AASD developmental exposure protocols that most likely produce negative neuropathological and functional outcomes. The experimental design includes a comparison of the effects resulting from a single, putatively nontoxic exposure to a prototypical anesthetic agent, with those accruing from repeated exposures of the same agent on levels of apoptotic degeneration in the developing brain and on subsequent behavioral functions. Specifically, we exposed mouse pups to isoflurane (ISO) at one or multiple ages and quantified the acute apoptotic response. In addition, we characterized the effects of repeated exposures to ISO at very young postnatal ages (P3+5+7) on a variety of behavioral functions including learning and memory, motor/sensorimotor capabilities, emotionality, and social interactions. Our behavioral studies also incorporated an analysis of sex-related effects since this variable has received little attention within the research area^16^.

## Results

### Neonatal ISO exposures induce elevated levels of neuroapoptosis in mice

**Figure 1:**
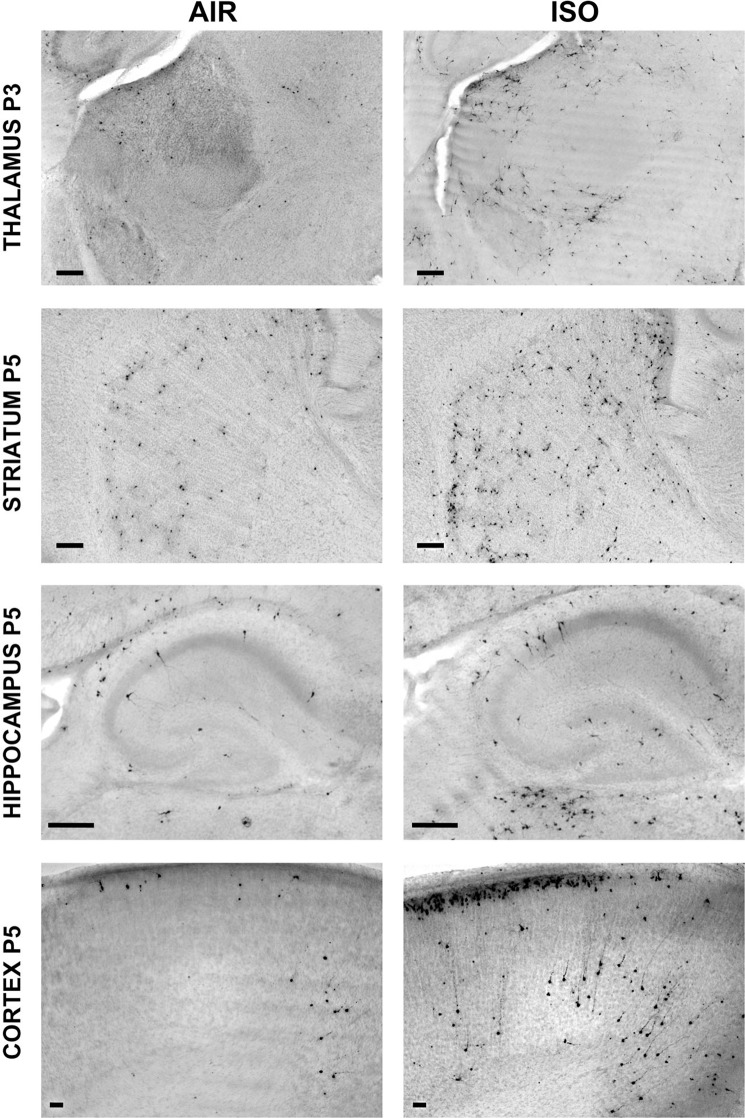
Age at greatest vulnerability to increased apoptosis induced by ISO in the developing mouse brain is region-specific. Mouse pups were exposed to 1.5% ISO or AIR only for 3 h on P3, P5, or P7, and processed for AC3+ IHC beginning 6 h after initiation of exposure. Representative AC3+ IHC images of mouse pup brain at the ages at which the greatest increase in apoptotic neurodegenerative response induced by ISO above typical developmental levels (AIR) was observed. Thalamus, P3; Hippocampus, P5; Striatum, P5; Cortex; P5. Scale bar is 50 µm.

**Figure 2:**
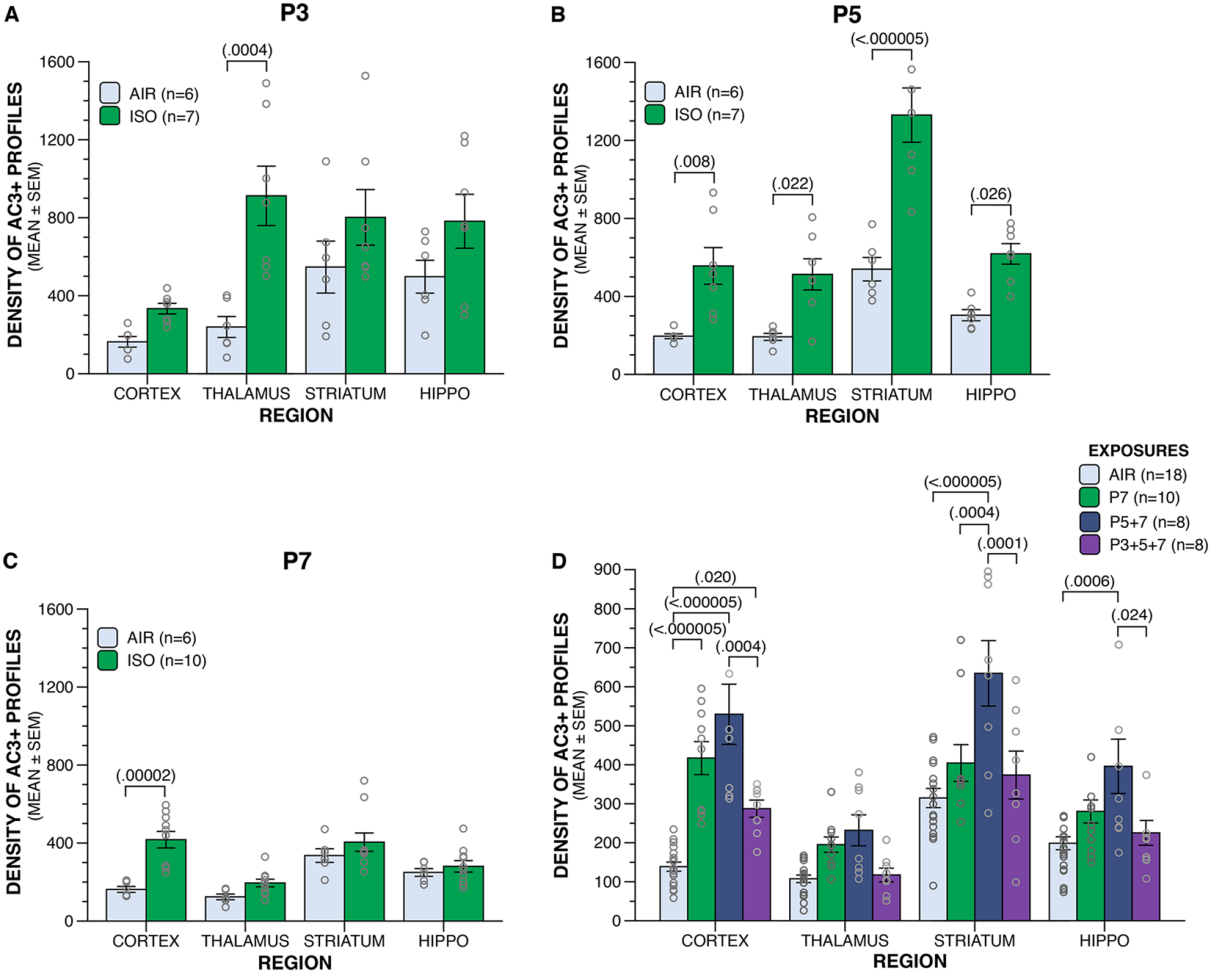
Neonatal ISO exposure increased the density of AC3+ profiles following single and multiple exposures in an age-dependent manner. Mouse pups were exposed to 1.5% ISO for 3 h or AIR only on P3, P5, P7, P5+7 or P3+5+7 and the density of AC3+ profiles was quantified in four distinct brain regions. (**A**) At P3, increased AC3+ densities were observed in the thalamus only of ISO-exposed brains compared to AIR-exposed controls. (**B**) At P5, an increase in AC3+ densities was observed in all brain regions examined. (**C**) At P7, increased AC3+ densities were observed only in the cortex of ISO-exposed brains compared to AIR-exposed controls. (**D**) In the cortex, enhanced AC3+ densities were observed following ISO exposure on P7, P5+7, and P3+5+7 compared to AIR only exposure. No change in AC3+ density over AIR controls was observed in the thalamus at P7 regardless of number of exposures. Only following ISO exposure on P5+7 were increased AC3+ densities observed in the striatum and hippocampus over AIR controls. Means ± SEM are shown. All data points represented by open gray circles.

Following published methods regarding ISO inhalational exposure in rodents^43^, C57BL/6 pups were exposed to 1.5% ISO or oxygenated air (AIR) for 3 h at P3, P5, P7 or P3+5+7. Acute neuroapoptotic response was assessed using activated-caspase-3 (AC3) immunohistochemistry (IHC) with degrees of neurodegeneration quantified by the optical dissector and fractionator method^44^. Exposure of neonatal mice to a single dose of ISO induced abnormally high levels of neuroapoptosis in the cortex, thalamus, dorsal striatum, and dorsal hippocampus in an age-dependent manner (Fig. 1; Table 1). ISO exposure at P3 significantly increased AC3+ profiles compared to AIR in the thalamus (Fig. 2A), in contrast to exposure at P5, when levels of AC3+ profiles were significantly increased in all brain regions (Fig. 2B). At P7, the cortex was the only brain region to show significantly increased neuroapoptosis for ISO relative to AIR (Fig. 2C). Thus, our single-age ISO data suggests the pattern and degree of drug-induced neuroapoptosis varies as a function of age, which may be related to the ontogenetic development of the structures involved^19^.

**Table 1:** Statistical results of AC3+ IHC quantification in brains of ISO- and AIR-exposed mouse pups.

Age At Exposure	rmANOVA interaction	Within Region Comparisons (Drug)			
	Region × Drug	Cortex	Thalamus	Striatum	Hippocampus
P3	*F*(3,33) = 3.313, *p* = 0.032	*F*(1,44) = 1.173, *p* = 1.000	*F*(1,44) = 18.289, *p* = 0.0004	*F*(1,44) = 2.622, *p* = 0.448	*F*(1,44) = 3.271, *p* = 0.308
P5	*F*(2.82,31.02) = 5.443, *p* = 0.005	*F*(1,44) = 10.746, *p* = 0.008	*F*(1,44) = 8.491, *p* = 0.022	*F*(1,44) = 51.644, *p* < 0.000005	*F*(1,44) = 8.179, *p* = 0.026
P7	*F*(3,42) = 4.644, *p* = 0.007	*F*(1,56) = 25.553, *p* = 0.00002	*F*(1,56) = 1.987, *p* = 0.656	*F*(1,56) = 1.851, *p* = 0.716	*F*(1,56) = 0.379, *p* = 1.000
P7, P5+7, P3+5+7	**Region × Exposure**	*F*(3,360) = 24.762, *p* < 0.000005	*F*(3,360) = 2.768, *p* = 0.176	*F*(3,360) = 13.851, *p* < 0.000005	*F*(3,360) = 5.518, *p* = 0.004
	*F*(5.89,78.5) = 4.280, *p* = 0.0009				

All distributions were normal and p-values reflect Bonferroni correction.

One of the main goals of this study was to investigate the behavioral effects of ISO on the developing brain. Since the human literature suggested that repeated, but not single, anesthetic exposures during childhood were associated with adverse developmental and behavioral outcomes^8–11^, we evaluated the effects of multiple administrations of ISO during the early neonatal period (P3-P7). To accomplish this goal, we conducted parallel studies in separate cohorts of mice exposed to 1.5% ISO or AIR for 3 h at P3+5+7: one cohort was used for histopathological studies while another cohort was used for behavioral analyses. The mice used for histopathology studies were euthanized 3 h following the final exposure at P7 and brain tissue was processed for AC3-IHC and quantification of AC3+ profiles as described above. The mice used in the behavioral cohort were studied on a battery of behavioral tests beginning in the early post-weaning period up into adulthood.

Analysis of the AC3+ profile data from the multiple ISO dosing study (Fig. 2D; Table 1) showed, in good agreement with results from the P7 group in the single ISO dosing study, that the cortex was the only brain region to have a significant increase in neuroapoptosis relative to the AIR group. Moreover, the cortex was the only region that showed significantly more AC3+ cells relative to AIR, regardless of the number of prior exposures (i.e., in the P5+7 and P3+5+7 ISO groups). However, AC3+ profiles were found to be significantly elevated in the cortex, striatum, and hippocampus in the P5+7 ISO group relative to their respective AIR groups. Interestingly, AC3+ profiles were significantly *decreased* in the cortex, striatum, and hippocampus of the P3+5+7 ISO groups relative to the P5+7 groups; although the apoptosis response in the hippocampus was less than that observed in these other two brain regions. We hypothesize that this effect is likely due to very large deletions of neurons resulting from previous ISO exposures, and the number of surviving neurons vulnerable to drug-induced apoptosis was greatly reduced. It is possible the reduced degree of apoptotic response observed within the hippocampus at P3+5+7 is attributable to the higher levels of neurogenesis in this area, and this may contribute to the decreased density of AC3+ profiles. Previous research suggests progenitor proliferation in the hippocampus is decreased for up to five days following neonatal ISO exposure^30^, although the role of perturbed neurogenesis and its interaction with apoptosis following neonatal anesthesia exposure remains to be evaluated in future studies^45^. It is noteworthy that the levels of AC3+ profiles were unaltered in the thalamus at P7 following the repeated ISO exposures. We interpret this lack of effect of multiple ISO doses on the thalamus to at least partially reflect that P7 is an age beyond the peak sensitivity of ISO-induced apoptosis in the thalamus.

### Multiple neonatal exposures of ISO induce only mild behavioral deficits

To investigate the possible longitudinal impact of ISO-induced increased neuroapoptosis response on behavioral function, we exposed male and female C57BL/6 mouse pups to ISO or AIR on P3+5+7, using the same exposure parameters as reported above. We assessed behaviors across multiple domains to characterize the influence of multiple ISO exposures on learning and memory and other behavioral functions not yet comprehensively examined in the rodent, including neuromotor, emotionality and social interactions. Our behavioral characterization was initiated during the juvenile stage and continued into adulthood (see Table 2 for order of and ages at testing). Based on the findings in previous rat studies suggesting important sex-specific behavioral consequences following neonatal ISO exposure^23,46^, behavioral performance of males and females was also analyzed separately to identify sex-dependent behavioral effects of neonatal ISO exposure in the mouse.

**Table 2:** Order of and age at testing to evaluate behavioral effects of multiple neonatal ISO exposures.

Behavioral Tests	Age At Testing
1- h locomotor activity	P31/P137
Sensorimotor battery	P32–33
Morris water maze	P34–42
Rotarod	P45, P49, P53
Elevated plus maze	P54–56/P144–146
Social approach	P60/P178
Conditioned fear	P194–196

To identify any gross developmental delay induced by multiple ISO exposures that might underlie long-term alterations in behavioral functions, we inspected the mice daily for any signs of gross physical abnormality and monitored body weight from P3 through weaning at P21. We did not observe physical anomalies in the treated mice and analysis of pre-weaning body weights showed the mice were unaffected by ISO exposure (Supplementary Table S1), suggesting multiple ISO exposures did not induce gross developmental delay. In addition, we did not observe weight differences between ISO and AIR mice at later ages corresponding to behavioral testing (P30, P45 and P60). These results thereby eliminate ISO-induced delays in growth and development as a confounding factor.

**Figure 3:**
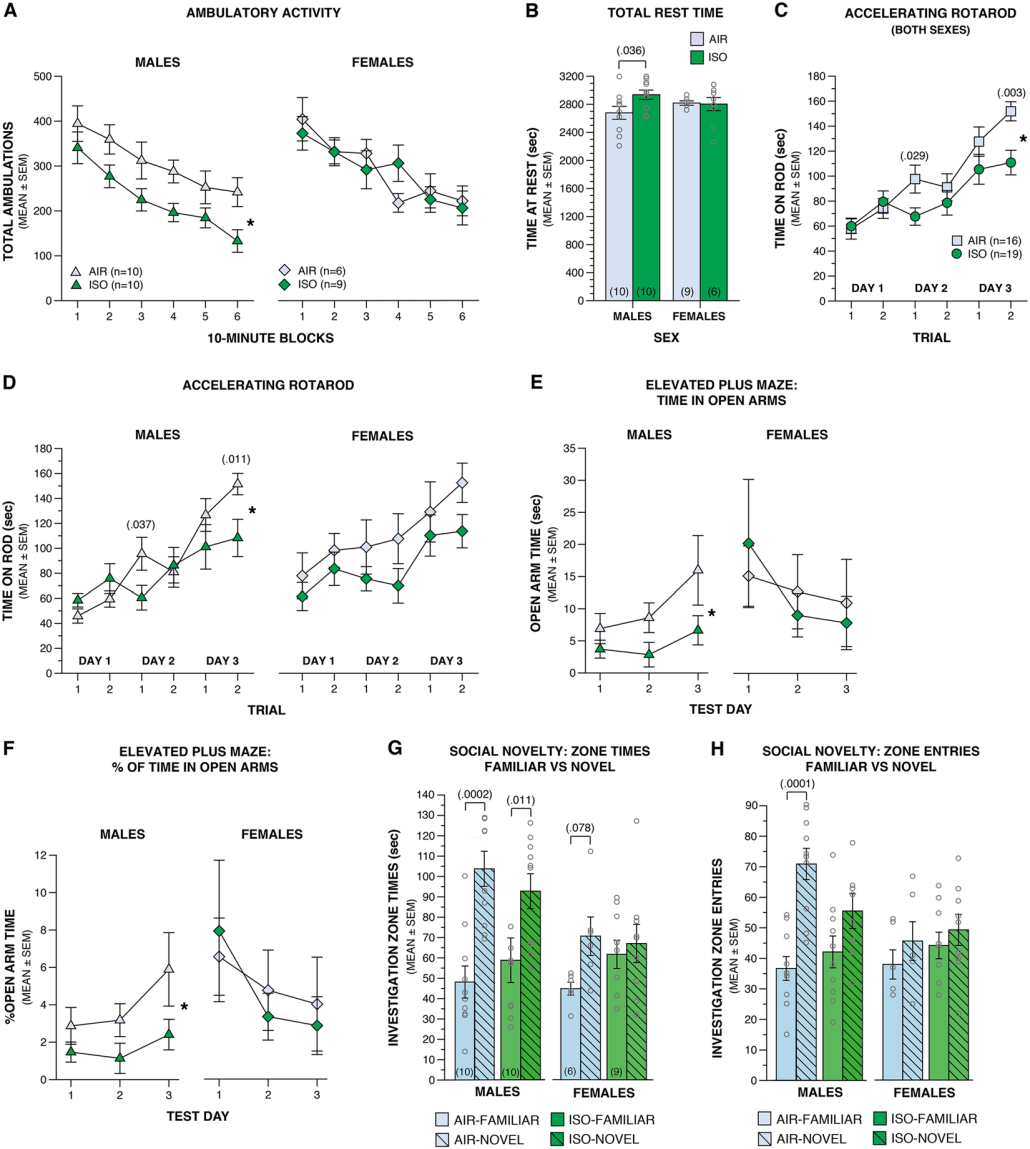
Multiple neonatal ISO exposures results in subtle, sex-specific behavioral disruptions. Male and female mice were exposed to 1.5% ISO for 3 h or AIR only on P3+5+7 and behaviorally characterized across multiple domains beginning during the juvenile stage and continuing into adulthood. (**A**,**B**) During the 1-h locomotor activity/exploration test, male, but not female, ISO mice exhibited (**A**) decreased total ambulations across the six 10-min blocks (*p = 0.031) and (**B**) increased total time at rest. (**C**) ISO mice spent less time on the accelerating rotarod during the first trial on Day 2 and second trial on Day 3 compared to AIR mice (*p = 0.003, Day 3). (**D**) These deficits were most pronounced between male mice (*p = 0.012, Day 3). (**E,F**) Male ISO mice spent less (**E**) absolute (*p = 0.028) and (**F**) percent total time in the open arms (*p = 0.032) of the EPM compared to male AIR mice. Female mice displayed comparable EPM performance across groups. (**G**) During the preference for social novelty trial of the social approach task, both male ISO and AIR mice exhibited a preference to spend more time investigating the novel conspecific and female AIR mice exhibited a trend for this preference, while female ISO mice spent a similar amount of time investigating both the familiar and novel conspecifics. (**H**) Only male AIR mice exhibited a greater number of entries into the investigation zone around the novel mouse compared to that around the familiar mouse. Means ± SEM are shown. All data points represented by open gray circles.

Functional characterization of ISO-exposed mice revealed subtle, sex-specific abnormalities across several behavioral domains. Behavioral assessment began with tests to measure general activity or sensorimotor disruptions that may interfere with interpretation of later behavioral test results. On P31, a 1-h locomotor activity/exploration test was used to assess general activity, exploratory behavior, and emotionality of the mice. Two days later (P32–33) a sensorimotor test battery was used to evaluate balance, strength, and coordination as previously described^47^. No differences between groups were observed for any of the sensorimotor tests for males or females (Supplementary Table S1). Also, although no significant overall effects for ISO were found for ambulatory activity or vertical rearing, sex-dependent differences were found with regard to general activity. Specifically, male ISO mice displayed significantly fewer total ambulations (whole body movements) on average across time compared to the male AIR control group (Fig. 3A; Table 3), although pair-wise comparisons did not reveal significant differences for any given time blocks. Similarly, while there were no significant overall drug effects for time spent at rest, the male ISO group showed significantly increased rest times compared to the male AIR mice (Fig. 3B; Table 3). No differences were observed in females regarding these two variables. In addition, there were no significant overall drug effects, nor any sex-specific differences found for vertical rearing or any emotionality (center of chamber) variables (Table 3).

**Table 3:** Statistical results of behavioral testing of ISO- and AIR-exposed mice.

Variable	Comparison	Statistical test	Output	P value	Nonparametric
Total ambulations (across 10-min blocks)	Drug	two-way rmANOVA	*F*(1,31) = 2.497	*p* = 0.124	normal
	Males only, Drug	rmANOVA	*F*(1,18) = 5.485	*p* = 0.031	normal
	Females only, Drug	rmANOVA	*F*(1,13) = 0.005	*p* = 0.952	normal
Rearing (across 10-min blocks)	Drug	two-way rmANOVA	*F*(1,31) = 2.625	*p* = 0.115	normal
	Males only, Drug	rmANOVA	*F*(1,18) = 0.447	*p* = 0.512	n/a
	Females only, Drug	rmANOVA	*F*(1,13) = 4.360	*p* = 0.057	normal
Time at rest	Drug	two-way ANOVA	*F*(1,31) = 2.100	*p* = 0.157	normal
	Male only, Drug	ANOVA	*F*(1,18) = 5.148	*p* = 0.036	normal
	Females only, Drug	ANOVA	*F*(1,13) = 0.017	*p* = 0.898	normal
Time in center area	Drug	two-way ANOVA	*F*(1,31) = 0.049	*p* = 0.823	normal
	Males only, Drug	ANOVA	*F*(1,18) = 0.162	*p* = 0.692	normal
	Females only, Drug	ANOVA	*F*(1,13) = 0.013	*p* = 0.911	normal
Entries into center area	Drug	two-way ANOVA	*F*(1,31) = 1.343	*p* = 0.255	normal
	Males only, Drug	ANOVA	*F*(1,18) = 4.072	*p* = 0.059	normal
	Females only, Drug	ANOVA	*F*(1,13) = 0.046	*p* = 0.883	*U*(15) = 21, *p* = 0.529
Accelerating rotarod (across 3 days/6 trials)	Drug × Day	two-way rmANOVA	*F*(1,62) = 3.153	*p* = 0.049	n/a
	Day 2, Drug	multiple comparison	*F*(1,186) = 5.774	*p* = 0.051	*U*(35) = 89, *p* = 0.037
	Day 3, Drug	multiple comparison	*F*(1,186) = 10.739	*p* = 0.003	*U*(35) = 92, *p* = 0.048
	Males only, Drug × Day	rmANOVA	*F*(2,36) = 4.648	*p* = 0.016	normal
	Males only, Day 2, Drug	multiple comparison	*F*(1,108) = 1.602	*p* = 0.208	*U*(20) = 36, *p* = 0.315
	Males only, Day 3, Drug	multiple comparison	*F*(1,108) = 8.594	*p* = 0.012	normal
	Females only, Drug × Day	rmANOVA	*F*(2,26) = 0.357	*p* = 0.703	normal
	Females only, Day 2, Drug	multiple comparison	*F*(1,78) = 3.946	*p* = 0.050	*U*(15) = 11, *p* = 0.066
	Females only, Day 3, Drug	multiple comparison	*F*(1,78) = 3.307	*p* = 0.073	normal
Time in open arm (across 3 days)	Drug	two-way rmANOVA	*F*(1,31) = 1.095	*p* = 0.303	n/a
	Males only, Drug	rmANOVA	*F*(1,18) = 5.750	*p* = 0.028	n/a
	Females only, Drug	rmANOVA	*F*(1,13) = 0.007	*p* = 0.934	normal
Percent time in open arm (across 3 days)	Drug	two-way rmANOVA	*F*(1,31) = 1.219	*p* = 0.278	n/a
	Day × Sex	two-way rmANOVA	*F*(1.95,60.28) = 3.481	*p* = 0.038	n/a
	Males only, Drug	rmANOVA	*F*(1,18) = 5.425	*p* = 0.032	n/a
	Females only, Drug	rmANOVA	*F*(1,13) = 0.024	*p* = 0.879	normal
Sociability, investigation zone time (social vs. empty)	Drug × Zone	two-way rmANOVA	*F*(1,31) = 0.010	*p* = 0.920	n/a
	AIR, Zone	planned comparison	*F*(1,31) = 51.581	*p < *0.000005	*Z* = −3.516, *p* = 0.0004
	ISO, Zone	planned comparison	*F*(1,31) = 67.629	*p* < 0.000005	normal
	AIR males only, Zone	planned comparison	*F*(1,31) = 41.580	*p* < 0.000005	*Z* = −2.201, *p* = 0.028
	AIR females only, Zone	planned comparison	*F*(1,31) = 16.726	*p* = 0.0003	normal
	ISO males only, Zone	planned comparison	*F*(1,31) = 38.926	*p* = 0.000001	normal
	ISO females only, Zone	planned comparison	*F*(1,31) = 29.340	*p* = 0.000007	normal
Sociability, investigation zone entries (social vs. empty)	Drug × Zone	two-way rmANOVA	*F*(1,31) = 1.165	*p* = 0.289	normal
	AIR, Zone	planned comparison	*F*(1,31) = 54.161	*p < *0.000005	normal
	ISO, Zone	planned comparison	*F*(1,31) = 44.190	*p* < 0.000005	normal
	AIR males only, Zone	planned comparison	*F*(1,31) = 43.581	*p < *0.000005	normal
	AIR females only, Zone	planned comparison	*F*(1,31) = 17.602	*p* = 0.0002	normal
	ISO males only, Zone	planned comparison	*F*(1,31) = 21.073	*p = *0.00007	normal
	ISO females only, Zone	planned comparison	*F*(1,31) = 23.118	*p* = 0.00004	normal
Social novelty, investigation zone time (familiar vs. novel)	Drug × Zone	two-way rmANOVA	*F*(1,31) = 2.882	*p* = 0.099	n/a
	Sex × Zone	two-way rmANOVA	*F*(1,31) = 5.545	*p* = 0.025	normal
	AIR, Zone	planned comparison	*F*(1,31) = 19.266	*p = *0.0001	normal
	ISO, Zone	planned comparison	*F*(1,31) = 5.662	*p* = 0.024	*Z* = −1.771, *p* = 0.077
	AIR males only, Zone	planned comparison	*F*(1,31) = 23.975	*p* = 0.00003	normal
	AIR females only, Zone	planned comparison	*F*(1,31) = 3.095	*p* = 0.088	normal
	ISO males only, Zone	planned comparison	*F*(1,31) = 8.939	*p* = 0.005	*Z* = −1.988, *p* = 0.047
	ISO females only, Zone	planned comparison	*F*(1,31) = 0.197	*p* = 0.660	normal
Social novelty, investigation zone entries (familiar vs. novel)	Drug × Zone	two-way rmANOVA	*F*(1,31) = 2.457	*p* = 0.127	normal
	Sex × Zone	two-way rmANOVA	*F*(1,31) = 5.462	*p* = 0.026	normal
	AIR, Zone	planned comparison	*F*(1,31) = 14.146	*p = *0.0007	normal
	ISO, Zone	planned comparison	*F*(1,31) = 3.493	*p* = 0.071	normal
	AIR males only, Zone	planned comparison	*F*(1,31) = 25.172	*p* = 0.00002	normal
	AIR females only, Zone	planned comparison	*F*(1,31) = 0.759	*p* = 0.390	normal
	ISO males only, Zone	planned comparison	*F*(1,31) = 3.864	*p* = 0.058	normal
	ISO females only, Zone	planned comparison	*F*(1,31) = 0.506	*p* = 0.482	normal
	Males only, Novel Zone, Drug	multiple comparison	*F*(1,62) = 5.173	*p* = 0.052	normal

For non-normal distributions, Mann-Whitney U and Wilcoxon Signed-Ranks nonparametric tests for two independent and paired samples, respectively, are also reported. Equivalent nonparametric test for two-way rmANOVA not available (n/a). Bonferroni correction reflected in p-values, where appropriate.

Because learning disabilities were linked to multiple anesthesia exposures in children^8,9^ and learning and memory deficits were observed previously in rodents following neonatal anesthesia exposure^23,29,30,33,46,48–51^, we evaluated our mice in the Morris water maze (MWM) to assess spatial learning and memory following our standard protocols^47^. Beginning on P34, MWM testing started with two days of cued trials to evaluate possible nonassociative dysfunctions induced by ISO that might compromise swimming ability. The mice then received acquisition training during five days of place trials, with a 60-s probe trial conducted 1 h after the last place trial on day 5 to evaluate retention of the platform location. No differences were observed between groups for male or female mice during the cued, place or probe trials (Supplementary Fig. S1, Supplementary Table S1). These data suggest that multiple ISO exposures on P3+5+7 did not produce demonstrable deficits in acquisition or retention capabilities on a spatial reference memory-based test in juvenile mice.

Mice were tested on the rotarod to assess the effects of ISO on fine motor coordination according to our standardized procedures^52^. The mice received 1 stationary rod trial, 2 constant rotational speed trials, and 2 accelerating rotational speed trials on P45, 49, 53. No significant overall effects involving drug were observed for the stationary rod or constant speed rotarod trials, nor were any sex-specific effects found during these procedures. However, analysis of the data from the accelerating rotarod trials yielded a significant Drug x Day interaction (Fig. 3C; Table 3), and a marginally nonsignificant Drug effect. Subsequent comparisons showed ISO mice remained on the rod for a significantly shorter duration compared to the AIR control group on day 3, according to the Bonferroni criterion (critical alpha *p* < 0.008), and large, yet marginally significant, differences were also observed for day 2. A major component of the differences observed on trial 2, day 3 were due to inferior performance by male ISO mice compared to the male AIR control group (Fig. 3D; Table 3). Deficits exhibited by the ISO mice on the accelerating rotarod reflect either impaired coordination or motor learning disturbances. Although our protocol was designed to minimize the effects of motor learning by separating test sessions by four intervening days, motor learning still readily occurred, thus making it difficult to separate motor performance from acquisition effects.

Anxiety-like behaviors were assessed in the elevated plus maze (EPM) for 3 consecutive days on P54–56, using our standardized procedures^47^. No significant overall effects involving Drug were found concerning time spent in the open arms (Table 3). However, planned analyses conducted on each sex revealed a significant Drug effect for the male groups indicating male ISO mice spent significantly less time in the open arms on average across test days compared to the male AIR mice (Fig. 3E; Table 3). However, pair-wise comparisons did not show any significant differences for any given test day, and no significant effects were found for the female groups (Fig. 3E; Table 3). We also examined the percent of total time spent in the open arms out of the total time spent in both sets of arms (% open arm time) to help control for confounding influences due to differences in activity levels between groups. Analysis of these data yielded a significant Sex x Test Day interaction for % open arm time, while planned analyses according to sex revealed results similar to those found for open arm time. Specifically, a significant Drug effect was observed for the male groups showing that, on average across test days, the male ISO mice spent significantly lower percentage of time in the open arms compared to the male AIR controls (Fig. 3F; Table 3). Again, no significant pair-wise comparisons were observed for any given test day, and analysis of the female groups failed to demonstrate any significant effects (Fig. 3F; Table 3).

Sociability and preference for social novelty were assessed on P60 through the use of our standardized social approach procedure^47^, which was developed with minor adaptation from methods previously described^53–56^. As defined in this measure of restrictive interactions, sociability is reflected by increased social investigation of a novel conspecific over an empty withholding cage, while preference for social novelty is indicated by increased social investigation of a novel versus familiar conspecific. No chamber or zone biases were observed during the habituation trial for either group of males or females, suggesting these variables were not confounding influences on investigatory behavior. ISO and AIR control mice of both sexes each displayed robust sociability in terms of spending a greater time in the investigation zone surrounding the social stimulus (conspecific) compared to the zone time for the non-social stimulus (Table 3). The same results were found for entries made into the investigation zones surrounding the social and non-social stimuli (Table 3). During the preference for social novelty trial, male ISO and AIR control mice spent more time in the investigation zone surrounding the novel mouse relative to the time spent in the zone surrounding the familiar mouse (Fig. 3G; Table 3). Among female mice, neither the ISO nor the AIR groups displayed a significant preference for the novel versus the familiar mouse, although the AIR controls showed a trend toward spending more time in the investigation zone surrounding the novel conspecific compared to the zone times observed for the familiar mouse (Fig. 3G; Table 3). The failure to reach statistical significance in the female control group may be due to the reduced power resulting from the low sample size (n = 6). Results from analyzing the investigation zone entries data were similar, except that male AIR mice exhibited significantly more entries into the novel mouse zone compared to the familiar mouse zone, while the ISO males did not (Fig. 3H; Table 3). The male AIR group also showed a nonsignificant trend toward more entries into the zone surrounding the novel mouse compared to entries of the ISO males (Fig. 3H; Table 3). No differences in entries between investigation zones were observed for female groups (Fig. 3H; Table 3).

To determine whether sex-dependent deficits in the ISO mice described above changed with age, we retested both groups of mice later in adulthood (P137-P178) using the locomotor activity, EPM and social approach tests. Analysis of the ambulatory activity data from the re-test revealed a significant Drug x Sex x Time interaction (Supplementary Table S2), although additional contrasts involving males and females and subsequent pair-wise comparisons did not show significant differences between groups. Analysis of the data from the EPM and social approach retest did not reveal any significant effects involving Drug or any sex-dependent effects (Supplementary Table S2). Lastly, we also assessed possible deficits in Pavlovian conditioning by evaluating the mice on our conditioned fear procedure^52^. No significant overall effects involving Drug or any sex-dependent effects were found with regard to freezing levels observed during a two-min baseline period or during CS-US training on day one, during the contextual fear test on day two, or on the altered context baseline or auditory cue test on day three (Supplementary Fig. S1; Supplementary Table S1).

## Discussion

To elucidate the acute neurological and functional impact of repeated exposures to a prototypical general anesthetic during synaptogenesis, we repeatedly exposed mouse pups to ISO and examined the acute neuroapoptotic response and long-term behavioral responses across several domains. As expected, the degree of drug-induced neuroapoptosis varied according to brain region. The thalamic response peaked earliest at P3, the hippocampus and striatum around P5, and the cortex remained vulnerable through P7. These results are consistent with temporal and spatial patterns of neuroapoptosis susceptibility shown in previous studies^16,17^. Our histopathological analysis suggests the striatum and cortex are the most vulnerable to repeated ISO exposure because these areas showed additional drug-induced apoptosis with subsequent exposures. Abnormally increased neuroapoptosis was observed only in the cortex following three ISO exposures. The neuroapoptotic response was greatly reduced in other areas examined, likely due to large deletions of neurons having occurred from previous exposures.

Our results provide additional information about the risk conferred by multiple anesthesia exposures for inducing possible functional disturbances. We evaluated the behavioral consequences of exposure to a prototypical general anesthetic at early neonatal ages, and also included sex differences in our analyses. Specifically, we observed hypoactivity and increased anxiogenic behavior in males repeatedly exposed to ISO, as well as motor coordination disruptions in both sexes. Social preference behavior was impacted only in females. The impairments observed were mild compared to those found following more neurotoxic agents such as anesthetic cocktails^29^, high doses of ethanol^57^, or multiple doses of PCP^58^. Of note, we did not observe any learning and memory deficits. This was unexpected given the large number of studies reporting learning and memory impairments following AASD drug exposure in rodents^16^. It is possible the large amount of handling during development, i.e. daily weight assessments P3 – P21, masked our ability to observe deficits in these mice by providing enrichment during the pre-weaning period^57,59,60^. Re-testing our mice failed to replicate the findings observed in the younger mice. This may be due, again, to enriching effects of extensive handling and behavioral testing of the mice throughout their early lives. We have reported a similar effect in mice following neonatal exposure to high doses of ethanol where drug-treated mice exhibited profound spatial learning and memory deficits in the MWM as juveniles, but performed similarly to control mice when tested again in young adulthood^57^. An independent cohort tested only in young adulthood again displayed MWM deficits^57^. We have also reported the presence of spatial learning deficits in 4-month old rats following neonatal exposure to an anesthetic cocktail^29^, suggesting the potential to recover cognitive functions following exposure to AASD agents during development may depend on the magnitude of apoptotic degenerative response and degree of initial behavioral impairment. If the experience of extensive handling and behavioral testing have enriching effects on recovery of behavioral functions, subtle behavioral deficits resulting from neonatal administration of anesthetics might be mitigated throughout a study as a function of increasing experience with these parameters.

Considering the results from the present study, an appropriate next step might be to test an independent cohort without extensive handling during development or prior behavioral testing. We recently recommended this approach in a review of the use of animal models to evaluate functional consequences of anesthesia during early development^16^. Nevertheless, we decided against attempting to replicate our results with an independent cohort because of the relatively small effect sizes we observed and due to the infrequent clinical use of ISO currently. While preclinical research into the effects of ISO increases our knowledge about the functional consequences of exposure to a prototypical general anesthetic during development, and is thus important, we feel a more appropriate use of our resources is to design and conduct additional studies using more up-to-date AASD protocols such as those that involve sevoflurane or desflurane alone or in combination with other AASDs.

Results from the few studies that have examined the sex-specific impact of neonatal ISO exposure on later behavioral function in the rat are inconsistent, likely due to concentration/duration of ISO used. Consistent with increased vulnerability in males observed in our study, impaired learning and memory performances in males but not females were reported for novel object recognition, contextual fear conditioning, RAM, and MWM^46,50,51^. Lee *et al*. reported male rats exposed to ISO displayed deficits in social recognition (memory) whereas female rats were unaffected^46^. Our data, together with findings from these previous studies, suggest that early exposure to AASD drugs may result in sex-specific neurobehavioral outcomes and that biological sex should be a variable included in future AASD animal research.

Of considerable note to interpreting our present ISO results are recent findings suggesting that mouse and NHPs might be similarly affected by multiple ISO exposures in terms of behavioral impairments outside the domains of learning and memory. Specifically, rhesus macaques were exposed to ISO on one or three early postnatal days^42^, and when tested in infancy, the monkeys exposed to ISO repeatedly displayed disrupted motor reflexes and decreased activity during a free play test. When tested later in development (i.e., 12 months old), monkeys exposed to ISO across multiple days displayed increased affiliative and anxiety-related behaviors. These findings parallel those we observed in our mouse model of multiple neonatal ISO exposures: decreased activity levels, impaired motor coordination, increased anxiety-related behavior and subtle social preference disruptions.

Other noteworthy commonalities between species are underscored by results from NHP studies on the apoptotic effects of exposure to AASDs in the infant rhesus macaque across development, which have demonstrated patterns of regional vulnerability similar to those observed in rodents, with neuroapoptosis peaking at early ages in subcortical regions and lasting into later ages in the neocortex^12,17,61–64^. Functional consequences attributed to the neuroapoptotic response following exposure to AASDs during development may also involve apoptosis of glia, particularly oligodendrocytes, extending the age of vulnerability to this drug-induced toxicity beyond that observed for neurons^62–65^. Oligoapoptosis is markedly greater than neuroapoptosis in the NHP during later^65^, as opposed to earlier^64^, stages of development. We recently provided preliminary evidence that anesthetic exposure might also induce enhanced oligoapoptosis in the rodent^16^. While peak oligodendrocyte maturation likely occurs at later ages than when the mice were treated in the present study, it is possible oligoapoptosis played some role in disrupted function, as seen in the NHP^42,65^. It is also possible that the more severe behavioral phenotypes observed with exposures at later ages than used here may reflect an influence from enhanced oligoapoptosis. Although beyond the scope of the present study, our recent qualitative analysis of oligoapoptosis in P14–15 mice^16^ supports the need to investigate a window of vulnerability mediating oligo- versus neuro-apoptosis throughout rodent development. Such research would advance our understanding of how age- and cell-specific apoptotic events following AASD exposure might contribute to neurobehavioral outcomes.

In conclusion, we found that exposure to the general anesthetic ISO during early neonatal ages results in substantial acute apoptotic degeneration and that multiple exposures induce subtle sex-dependent effects on behavior. More work is needed to further clarify the mechanisms underlying AASD-induced sex-specific outcomes, and the role of cell-type-specific enhanced apoptosis in disrupting behavioral circuits. Identification of specific circuit disruptions will lead to novel adjunctive strategies that may help ameliorate the potential acute cell loss and long-term functional disruptions induced by AASD exposure in the developing brain.

## Methods

### Animals

All experimental protocols were approved by and performed in accordance with the relevant guidelines and regulations of the Institutional Animal Care and Use Committee of Washington University. Experimentally naïve C57BL/6 mouse pups were used from 15 litters for acute neuroapoptotic histopathology, 5 independent litters, 15 females and 20 males, for long-term behavioral analyses. The colony room lighting was 12:12 h light/dark cycle; room temperature (~20–22 °C) and relative humidity (50%) controlled automatically. Standard lab diet and water was freely available. Pregnant dams were individually housed in translucent plastic cages measuring 28.5 × 17.5 × 12 cm with corncob bedding. Upon weaning at P21, mice for behavioral testing were group housed according to sex and random distribution across treatment groups. All mice were carefully monitored throughout the experiment for weight and general appearance.

### Anesthesia exposure

Age- and litter-matched mouse pups were exposed to 1.5% ISO USP (Butler Animal Health Supply, Dublin, OH) or oxygenated air only (AIR), for 3 h at P3, P5, P7 or P3+5+7 following established methods^43^ with normoxic and normothermic conditions maintained. The concentration and duration of ISO exposure were chosen based on previous pilot studies demonstrating 1.5% for 3 h induced an apoptotic response without high mortality or excessive weight loss, and because it is substantially below that which has been cited as the MAC for the infant mouse (2.26% ISO)^66^. Oxygen was flowing at 30% for 6 liters/min. Pups were placed back with dam once consciousness was regained, and the nest was monitored for restored maternal care.

### Tissue processing, immunohistochemistry, and density quantification

Six h after initial exposure to ISO or AIR, pups were deeply anesthetized with sodium pentobarbital (i.p.) and perfused with a heparinized saline flush followed by 4% paraformaldehyde in a sodium phosphate buffer. Immediately following perfusion, the whole brain was removed and post-fixed in perfusate for 4 days, then serially sectioned (70 µm) on a Vibratome. AC3-IHC processing and quantification of AC3 positive profiles were conducted as previously described^44^. Sections were quenched in methanol containing 3% hydrogen peroxide, then washed in PBS, incubated in blocking solution (2% bovine serum albumin + 0.2% dry milk in 0.1% triton-X PBS) for 60 min, and then incubated overnight in rabbit anti-AC3 antiserum (1:1000; Cell Signaling Technology, Beverly, MA). The next day, sections were washed in PBS, incubated for 60 min in goat anti-rabbit secondary antiserum (1:200; Vector Labs, Burlingame, CA), and washed in PBS. Sections were then reacted in the dark with ABC reagents (Vectastain ABC Elite Kit, Vector Laboratories, Burlingame, CA) for 60 min, washed in PBS, and the color developed with VIP reagents (Vector VIP substrate kit for peroxidase, Vector Labs, Burlingame, CA). Finally, sections were dehydrated with a series of graded ethanols and cleared in Citrisolv before coverslipping with Permount. For apoptotic quantification, Stereo Investigator 7 (MBF Bioscience, Inc., Williston, VT) on a Pentium III PC, connected to a Prior Optiscan motorized stage (ES103 XYZ system, Prior Scientific Inc., Rockland MA) mounted on a Nikon Labophot-2 microscope was used. The boundaries of the brain regions of interest were manually traced, then AC3+ profiles were marked and the area of each region was calculated with reference to a standard atlas. To estimate the total number of AC3+ profiles in each brain region, regional counts were summed and then divided by the total area counted, and a mean density score was derived for each animal.

### Behavioral tests

All behavioral testing was conducted during the light cycle, by a female experimenter blinded to experimental group. All equipment was cleaned with 2% chlorhexidine diacetate or 70% ethanol between animals.

#### Locomotor activity

The mice were evaluated over a 1 h period in transparent enclosures (47.6 × 25.4 × 20.6 cm) surrounded by metal frames containing 4 × 8 matrices of photobeam pairs as previously described^47^. Computer software (MotorMonitor, Hamilton-Kinder, LLC, Poway, CA) quantified horizontal and vertical beam breaks as ambulations and rearings, respectively, in a 33 × 11 cm central zone and a bordering 5.5 cm peripheral zone. General activity variables (total ambulations, rearings, time at rest) along with measures of emotionality, including time spent, distance traveled and entries made into the central zone were analyzed.

#### Sensorimotor battery

Walking initiation, ledge, platform, pole, and inclined and inverted screen tests were performed as previously described^67^. Time in each task was manually recorded. The average for two trials was used for analyses. To avoid exhaustion effects, the order of the tests during the first set of trials was reversed for the second set of trials. Tests lasted 60 s, except for the pole test, which lasted 120 s. For walking initiation, time for an animal to leave a 21 × 21 cm square on a flat surface was recorded. For ledge and platform tests, the time the animal was able to balance on an acrylic ledge (0.75 cm wide and 30 cm high), and on a wooden platform (1.0 cm thick, 3.0 cm in diameter and elevated 47 cm) was recorded, respectively. The pole test was used to evaluate fine motor coordination by quantifying time to turn 180° and climb down a vertical pole. The screen tests assessed a combination of coordination and strength by quantifying time to climb up or hang onto a mesh wire grid measuring 16 squares per 10 cm, elevated 47 cm and inclined (60° or 90°) or inverted.

#### Morris water maze

MWM was conducted as previously described^47^. Briefly, cued, place and probe trials were conducted in a galvanized steel pool, measuring 120 cm in diameter, and filled with opaque water. The PVC escape platform measured 11.5 cm in diameter. A digital video camera connected to a PC computer and the computer software program ANY-maze (Stoelting Co., Wood Dale, IL) tracked the swimming pathway of the mouse to the escape platform and quantified path length, latency to find escape platform, and swimming speeds.

On two consecutive days, animals received four cued trials, separated by 1 h, during which a red tennis ball atop a rod was attached to the escape platform and served as a visual cue. To prevent spatial learning, the escape platform was moved to a different quadrant location for each trial. The mouse was released from the quadrant opposite to the platform location, allowed 60 s to locate the platform, and then allowed to sit on it for 30 s before being returned to its home cage. Three days later, animals received two blocks of two consecutive place trials on five consecutive days, with an intertrial interval between 30–90 s and approximately 2 h separating trial blocks. The escape platform remained in the same quadrant location for all place trials and distal cues were placed on the walls of the room to support spatial learning. The mouse was released from a different location for each trial on each day. The mouse was allowed 60 s to find the escape platform, and allowed to sit on it for 30 s before being returned to its home cage. Cued and place trials were combined into blocks of two or four trials for analyses, respectively. Immediately following completion of place trials, one 60 s probe trial was conducted to assess memory retention of the escape platform location, which was removed from the pool yet the distal cues remained.

#### Rotarod

For the rotarod (Economex, Columbus Instruments, Columbus, OH), animals were placed on a 9.5 cm section of grooved rod measuring 3.81 cm in diameter surrounded by plastic walls and elevated 52 cm from the floor. Animals received five trials on three test days separated by four days to minimize motor learning. The rod was stationary for trial 1 and continuously rotating at 2.5 rpm for the trials 2 and 3 for 60 s. The rod accelerated by 0.13 rpm per second for trials 4 and 5 for 180 s. Time the animal remained on the rod was used for analyses.

#### Elevated plus maze

EPM was conducted as previously described^47^. Briefly, a 5 min trial was conducted on each of 3 consecutive days. Animals were placed on a dimly lit black acrylic surface measuring 5 × 5 cm and elevated 63 cm above the floor equipped with photo beam pairs. Four arms (35 cm long, 5 cm wide; two open and two with 15 cm high walls) extended from a central area. The MotorMonitor software (Kinder Scientific, LLC, Poway, CA) quantified beam breaks as duration, distance traveled, entries, and time at rest in each zone (closed arms, open arms and center area).

#### Social approach

As previously described^47^, social approach apparatus was evaluated in a rectangular acrylic box divided into three separate chambers each measuring 19.5 × 39 × 22 cm including dividing walls with 5 × 8 cm doorways to allow for movement between chambers. Each outer chamber contained a stainless steel conspecific cage (inverted Galaxy Pencil Cup, Spectrum Diversified Designs, Inc, Streetsboro, OH) with vertical bars to allow minimal contact, measuring 10 cm in base diameter. A digital video camera and ANY-maze software (Stoelting Co., Wood Dale, IL.) tracked behavior of the mouse and time spent in 2 cm investigation zone surrounding the conspecific cages. Four consecutive 10 min trials were conducted. The first two trials served as apparatus habituation (trial 1, middle chamber only; trial 2, all three chambers and empty conspecific cages). During trial 3 (sociability), a sex- and age-matched conspecific was placed in one cage with the other empty. During trial 4 (social novelty preference), the now familiar conspecific remained, and an unfamiliar sex- and age-matched conspecific was placed in the other cage. Placement of conspecifics was counterbalanced between exposure groups.

#### Fear conditioning

All mice were evaluated for fear conditioning as described previously^52^. Briefly, animals were habituated to and tested in acrylic chambers, measuring 26 × 18 × 18 cm with a metal grid floor, an LED light bulb and an inaccessible peppermint odorant. Day 1 testing lasted 5 min, and an 80 dB tone sounded for 20 s at 100 s, 160 s and 220 s, paired with a 1.0 mA shock during the last 2 s of the tone. The freezing behavior was quantified through the computerized image analysis software program FreezeFrame (Actimetrics, Evanston, IL). Freezing was defined as no movement except for normal respiration. Day 2 lasted 8 min, and the light was illuminated yet no tones or shocks were presented to evaluate freezing behavior in response to the contextual cues associated with the shock from day 1. Day 3 lasted 10 min and the context of the chamber was changed to an opaque acrylic, walled chamber containing a coconut odorant. Baseline freezing behavior to the new context was quantified during the first 2 min. The 80 dB tone sounded 120–600 s. Freezing behavior to the auditory cue associated with the shock stimulus from day 1 was quantified. Shock sensitivity was evaluated following testing as previously described^52^.

### Statistical Analyses

All statistical analyses were performed using IBM SPSS Statistic software (v.24). Prior to analyses, all data were screened for fit of distributions with assumptions underlying univariate analyses, which included the Shapiro-Wilk test and qqplot investigations for normality. Means and standard errors were computed for each measure. Analysis of variance (ANOVA), including repeated measures, were used to analyze the histological and behavioral data. For social approach data, a priori planned comparisons were used. With a statistically significant interaction between main factors, simple main effects were calculated to provide clarification of statistically significant between-treatment and within-treatment differences. Where appropriate, the Huynh-Feldt adjustment was used to protect against violations of sphericity, the Bonferroni correction was applied to multiple pairwise comparisons, and Tukey’s HSD method was used as the post hoc test. Statistical results were confirmed with two-tailed non-parametric testing, when available, for any datasets with violations of the univariate assumptions. As the ANOVA is robust to violations of normality^68^, we are confident in our results and feel the ANOVA provides a more sensitive and powerful statistical test. However, we included non-parametric test results in the statistical tables where appropriate. The critical alpha value for all analyses was *p* < 0.05, unless otherwise stated. Test statistics and other details for each analysis are provided in Tables 1, 3, S1 and S2.

## Supplementary information

Supplementary Information

## Data Availability

The datasets generated during and/or analyzed during the current study are available from the corresponding author upon reasonable request.
