# Supplementary material for: Repeated neonatal isoflurane exposures in the mouse induce apoptotic degenerative changes in the brain and relatively mild long-term behavioral deficits
Source: Sci Rep. 2019 Feb 26;9:2779. doi: 10.1038/s41598-019-39174-6 (PMC6391407; doi:10.1038/s41598-019-39174-6)
Supplement: Supplementary file 1 — Supplementary Information [file 41598_2019_39174_MOESM1_ESM.pdf]

Supplementary Information

Repeated neonatal isoflurane exposures in the mouse induce apoptotic degenerative changes in the brain and relatively mild long-term behavioral deficits.

Susan E. Maloney, Carla M. Yuede, Catherine E. Creeley, Sasha L. Williams, Jacob N. Huffman, George T. Taylor, Kevin N. Noguchi, David F. Wozniak

Supplementary Table S1. Statistical results of behavioral testing of P3+5+7 ISO- and AIR-exposed mice.

VARIABLE	COMPARISON	STATISTICAL TEST	OUTPUT	P VALUE	NONPARAMETRIC
Body weights	Age x Drug interaction (P3, P4, P6, P8, P14, P21)	two-way rmANOVA	*F*(1.32,40.96)=0.716	*p* =.612	n/a
	Drug (P30)	two-way ANOVA	*F*(1,31)=0.007	*p* =.935	normal
	Drug (P45)	two-way ANOVA	*F*(1,31)=0.230	*p* =.635	normal
	Drug (P60)	two-way ANOVA	*F*(1,31)=0.669	*p* =.420	normal
Cued, path length (across 4 blocks of trials)	Drug	two-way rmANOVA	*F*(1,31)=0.093	*p* =.763	normal
	Trial	two-way rmANOVA	*F*(2.59,80.38)=25.454	*p*< .000005	normal
	Males only, Drug	rmANOVA	*F*(1,18)=0.066	*p* =.800	n/a
	Females only, Drug	rmANOVA	*F*(1,13)=0.391	*p* =.543	normal
Cued, latency to platform (across 4 blocks of trials)	Drug	two-way rmANOVA	*F*(1,31)=0.099	*p* =.755	normal
	Trial	two-way rmANOVA	*F*(2.64,81.67)=29.064	*p*< .000005	normal
	Males only, Drug	rmANOVA	*F*(1,18)=0.388	*p* =.541	normal
	Females only, Drug	rmANOVA	*F*(1,13)=0.612	*p* =.448	normal
Cued, swim speeds (across 4 blocks of trials)	Drug	two-way rmANOVA	*F*(1,31)=0.006	*p* =.939	normal
	Trial	two-way rmANOVA	*F*(1,93)=5.894	*p* =.001	normal
	Males only, Drug	rmANOVA	*F*(1,18)=0.324	*p* .576	normal
	Females only, Drug	rmANOVA	*F*(1,13)=0.202	*p* =.661	n/a
Place, path length (across 5 blocks of trials)	Drug	two-way rmANOVA	*F*(1,31)=0.320	*p* =.576	normal
	Trial	two-way rmANOVA	*F*(3.37,104.37)=12.152	*p*< .000005	normal
	Males only, Drug	rmANOVA	*F*(1,18)=0.124	*p* =.729	normal
	Females only, Drug	rmANOVA	*F*(1,13)=0.210	*p* =.654	normal
Place, latency to platform (across 5 blocks of trials)	Drug	two-way rmANOVA	*F*(1,31)=0.108	*p* =.744	normal
	Trial	two-way rmANOVA	*F*(3.68,114.19)=8.493	*p* =.000009	normal
	Males only, Drug	rmANOVA	*F*(1,18)=0.167	*p* =.687	normal
	Females only, Drug	rmANOVA	*F*(1,43)=0.002	*p* =.965	normal
Place, swim speeds (across 5 blocks of trials)	Drug	two-way rmANOVA	*F*(1,31)=0.066	*p* =.798	normal
	Trial	two-way rmANOVA	*F*(4,124)=20.016	*p*< .000005	normal
	Males only, Drug	rmANOVA	*F*(1,18)=0.263	*p* =614	normal
	Females only, Drug	rmANOVA	*F*(1,13)=0.893	*p* =.362	normal
Probe, target quadrant spatial bias	Drug x Quadrant interaction	two-way rmANOVA	*F*(1.64,50.70)=0.849	*p* =.413	normal
	Males only, Drug x Quadrant interaction	rmANOVA	*F*(1.77,31.98)=1.054	*p* =353	normal
	Females only, Drug x Quadrant interaction	rmANOVA	*F*(1.57,20.36)=0.771	*p* =.446	normal
Percent time freezing (across test minutes)	Day 1 baseline, Drug	two-way rmANOVA	*F*(1,31)=2.652	*p* =.114	n/a
	Day 1 shock+tone pairing, Drug	two-way rmANOVA	*F*(1,31)=1.141	*p* =.294	n/a
	Day 2 contextual fear, Drug	two-way rmANOVA	*F*(1,31)=1.047	*p* =.314	normal
	Day 3 baseline, Drug	two-way rmANOVA	*F*(1,31)=0.064	*p* =.802	n/a
	Day 3 auditory cued fear, Drug	two-way rmANOVA	*F*(1,31)=0.732	*p* =.399	normal

Equivalent nonparametric test for two-way rmANOVA not available (n/a) for non-normal distributions.

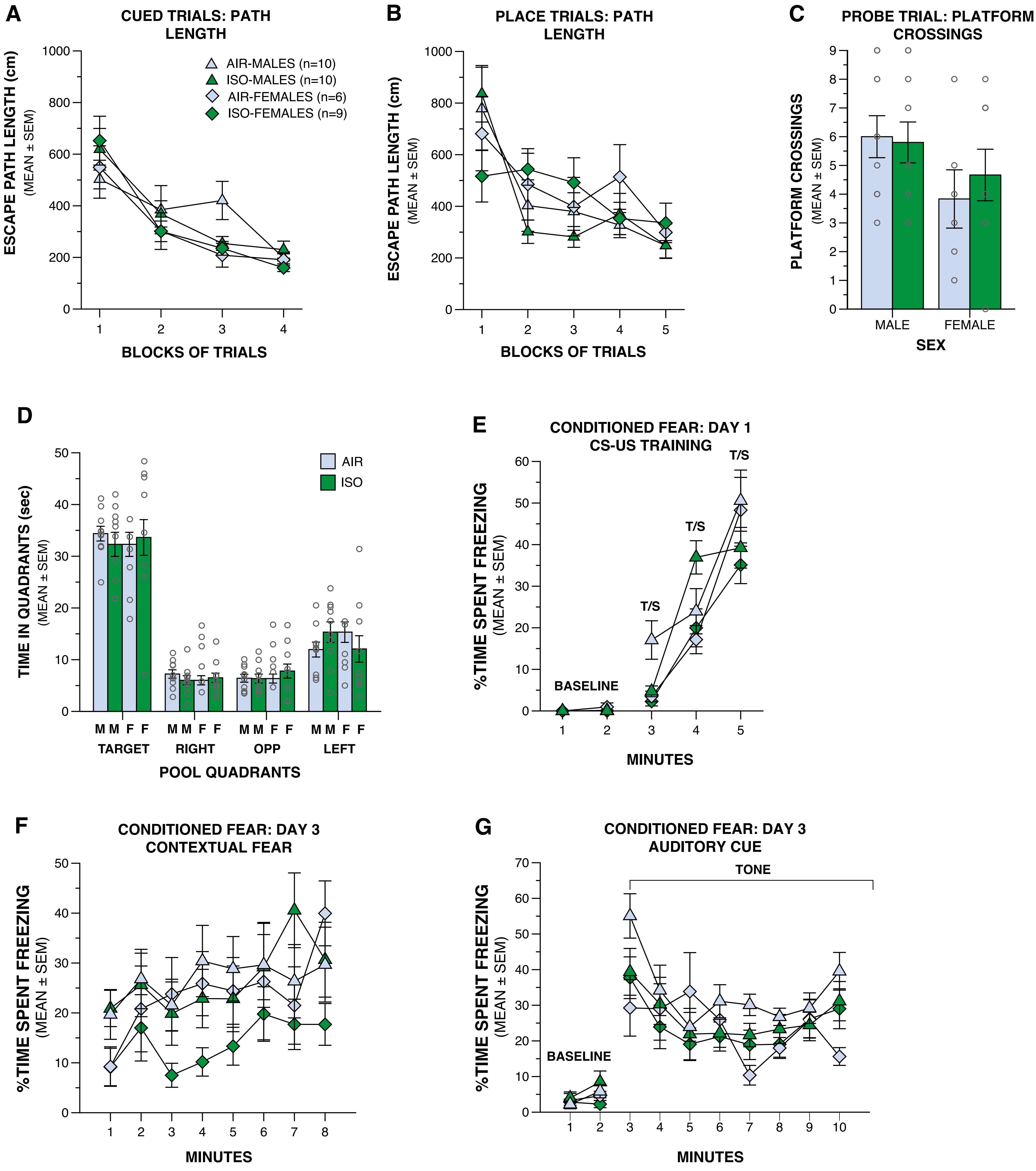

A
CUED TRIALS: PATH LENGTH
ESCAPE PATH LENGTH (cm)
(MEAN ± SEM)
AIR-MALES (n=10)
ISO-MALES (n=10)
AIR-FEMALES (n=6)
ISO-FEMALES (n=9)
BLOCKS OF TRIALS
B
PLACE TRIALS: PATH LENGTH
ESCAPE PATH LENGTH (cm)
(MEAN ± SEM)
BLOCKS OF TRIALS
C
PROBE TRIAL: PLATFORM CROSSINGS
PLATFORM CROSSINGS
(MEAN ± SEM)
MALE
FEMALE
SEX
D
TIME IN QUADRANTS (sec)
(MEAN ± SEM)
AIR
ISO
M M F F
TARGET
RIGHT
OPP
LEFT
POOL QUADRANTS
E
CONDITIONED FEAR: DAY 1 CS-US TRAINING
%TIME SPENT FREEZING
(MEAN ± SEM)
BASELINE
T/S
MINUTES
F
CONDITIONED FEAR: DAY 3 CONTEXTUAL FEAR
%TIME SPENT FREEZING
(MEAN ± SEM)
MINUTES
G
CONDITIONED FEAR: DAY 3 AUDITORY CUE
%TIME SPENT FREEZING
(MEAN ± SEM)
TONE
BASELINE
MINUTES

Supplementary Figure S1. Disruptions in learning and memory performance were not observed in mice exposed to neonatal ISO multiple times. Male and female mice were exposed to 1.5% ISO for 3h or AIR only on P3+5+7 and behaviorally characterized across multiple domains beginning during the juvenile stage and continuing into adulthood. **A-B)** ISO and AIR mice of both sexes traveled comparable path lengths to the platform during (**A**) cued and (**B**) place trials of the MWM. **C-D)** During the MWM probe trial, ISO and AIR mice of both sexes demonstrated (**C**) comparable platform location crossings and (**D**) spatial bias for the target quadrant containing the escape platform. **E-G)** ISO and AIR mice spent a comparable percent time freezing during the three days of fear conditioning: (**E**) pairing of shock + tone, (**F**) contextual fear, and (**G**) auditory cued fear. Means ± SEM are shown. All data points represented by open gray circles.

Supplementary Table S2. Statistical results of behavioral re-testing in later adulthood of P3+5+7 ISO- and AIR-exposed mice.

VARIABLE	COMPARISON	STATISTICAL TEST	OUTPUT	P VALUE	NONPARAMETRIC
Total ambulations (across 10-min blocks)	Drug x Sex x Block	two-way rmANOVA	$F(5,155)=2.626$	$p=.026$	normal
	Males only, Drug	rmANOVA	$F(1,18)=0.374$	$p=.549$	n/a
	Females only, Drug	rmANOVA	$F(1,13)=3.294$	$p=.093$	normal
Time in open arm (across 3 days)	Drug	two-way rmANOVA	$F(1,31)=0.009$	$p=.925$	normal
	Males only, Drug	rmANOVA	$F(1,18)=1.771$	$p=.200$	normal
	Females only, Drug	rmANOVA	$F(1,13)=0.767$	$p=.397$	normal
Percent time in open arm (Aross 3 days)	Drug	two-way rmANOVA	$F(1,31)=0.043$	$p=.837$	n/a
	Males only, Drug	rmANOVA	$F(1,18)=2.066$	$p=.168$	normal
	Females only, Drug	rmANOVA	$F(1,13)=0.687$	$p=.422$	normal
Sociability, investigation zone time (social vs. empty)	Drug x Zone	two-way rmANOVA	$F(1,31)=0.084$	$p=.774$	normal
	AIR, Zone	planned comparison	$F(1,31)=56.521$	$p<.000005$	normal
	ISO, Zone	planned comparison	$F(1,31)=78.950$	$p<.000005$	normal
	AIR males only, Zone	planned comparison	$F(1,31)=57.151$	$p<.000005$	normal
	AIR females only, Zone	planned comparison	$F(1,31)=13.350$	$p=.001$	normal
	ISO males only, Zone	planned comparison	$F(1,31)=45.654$	$p<.000005$	normal
	ISO females only, Zone	planned comparison	$F(1,31)=34.078$	$p<.000005$	normal
Social novelty, investigation zone time (familiar vs. novel)	Drug x Zone	two-way rmANOVA	$F(1,31)=0.846$	$p=.365$	normal
	AIR, Zone	planned comparison	$F(1,31)=28.285$	$p=.00001$	normal
	ISO, Zone	planned comparison	$F(1,31)=12.243$	$p=.001$	normal
	AIR males only, Zone	planned comparison	$F(1,31)=9.678$	$p=.004$	normal
	AIR females only, Zone	planned comparison	$F(1,31)=4.065$	$p=.053$	normal
	ISO males only, Zone	planned comparison	$F(1,31)=18.844$	$p=.0001$	normal
	ISO females only, Zone	planned comparison	$F(1,31)=10.304$	$p=.003$	normal

Equivalent nonparametric test for two-way rmANOVA not available (n/a) for non-normal distributions.
